# Unmasking the Rarity: A Case Report on Type B Lactic Acidosis in Pediatric Acute Lymphoblastic Leukemia

**DOI:** 10.7759/cureus.61201

**Published:** 2024-05-27

**Authors:** Keta Vagha, Atish Bakane, Aashita Malik, Chaitanya Kumar Javvaji, Sunita Vagha, Siddhartha Murhekar

**Affiliations:** 1 Pediatrics, Jawaharlal Nehru Medical College, Datta Meghe Institute of Higher Education and Research, Wardha, IND; 2 Oncology, Jawaharlal Nehru Medical College, Datta Meghe Institute of Higher Education and Research, Wardha, IND; 3 Pathology, Jawaharlal Nehru Medical College, Datta Meghe Institute of Higher Education and Research, Wardha, IND; 4 Trauma and orthopaedics, East Kent Hospitals University NHS Foundation Trust, Canterbury, GBR

**Keywords:** case report, hepatosplenomegaly, hematologic malignancy, metabolic complications, pediatric oncology, type b lactic acidosis, acute lymphoblastic leukemia

## Abstract

Acute lymphoblastic leukemia (ALL) is the most prevalent pediatric malignancy, accounting for approximately 25% of childhood cancers. Despite significant advancements in treatment protocols, ALL remains a complex disease, often presenting with various complications, including the rare metabolic disturbance of type B lactic acidosis. This case report details the clinical journey of a 14-year-old female with ALL who developed type B lactic acidosis during treatment. The patient presented with intermittent fever, abdominal pain, jaundice, and hepatosplenomegaly, accompanied by severe anemia and thrombocytopenia. Initial management included supportive care and chemotherapy initiation. Despite aggressive interventions, the patient's condition deteriorated, with escalating lactic acidosis and respiratory distress, leading to a critical need for tailored management strategies. This report underscores the importance of early recognition and comprehensive management of type B lactic acidosis in pediatric ALL, highlighting its multifactorial etiology and potentially life-threatening consequences. Enhanced clinical awareness and a multidisciplinary approach are crucial for improving outcomes in such complex cases.

## Introduction

Acute lymphoblastic leukemia (ALL) is the most prevalent malignancy among children, accounting for approximately 25% of pediatric cancers [1]. Despite significant advancements in treatment protocols, the disease remains complex, often presenting with various complications. Among these complications, lactic acidosis, specifically type B lactic acidosis, is an exceedingly rare metabolic aberration, particularly in pediatric ALL cases, and its underlying mechanisms remain poorly elucidated [2].

Lactic acid is a metabolic product generated during anaerobic glycolysis. The sole pathway for its removal from the body involves its conversion back to pyruvate through oxidation. Typically, the primary organs responsible for lactate elimination are the liver (accounting for 80-90% of the process) and the kidneys [3]. However, in pathological conditions, various mechanisms can escalate lactate production, disrupting the balance between its production and excretion, consequently leading to its accumulation in the body [4]. Serum lactate levels serve as a risk assessment marker and a therapeutic target. Elevated serum lactate levels, coupled with a prolonged duration for their normalization, correlate with an increased risk of mortality. Typical lactate concentrations fall below 2 millimoles/litre (mmol/L), while hyperlactatemia is delineated by lactate levels ranging from 2 mmol/L to 4 mmol/L. Lactate concentrations surpassing 4 mmol/L denote severe elevation [5].

Type B lactic acidosis is distinct from type A lactic acidosis, which typically arises from tissue hypoxia or ischemia. Instead, type B lactic acidosis is associated with underlying medical conditions such as hematologic malignancies, including leukemias and lymphomas, and often manifests without evidence of tissue hypoxia [6]. The occurrence of type B lactic acidosis in pediatric ALL patients presents considerable diagnostic and therapeutic challenges due to its multifactorial etiology and potentially life-threatening consequences.

In this comprehensive case report, we present a detailed account of a pediatric patient with ALL who developed type B lactic acidosis during the course of the treatment. 

## Case presentation

A 14-year-old female was admitted to our tertiary care hospital in central India with a one-month history of intermittent fever and abdominal pain, followed by bilateral leg pain for 15 days. On examination, she was pale, without palpable lymphadenopathy, and exhibited tachycardia (heart rate: 132 bpm) and tachypnea (respiratory rate: 38 breaths/min), with an oxygen saturation of 92% on room air and a blood pressure of 126/70 mmHg. Systemic abdomen examination revealed hepatomegaly (liver palpable 6 cm below the costal margin), splenomegaly (spleen palpable 5 cm below the costal margin), and mild diffuse abdominal tenderness, without other systemic abnormalities.

Initial laboratory investigations showed a hemoglobin level of 6.3 g/dl, total leukocyte count of 8000/cumm, platelet count of 74,000/cumm, total bilirubin of 1.5 mg/dl (unconjugated: 1.1 mg/dl, conjugated: 0.4 mg/dl), lactate of 1.8 mmol/L, and normal renal function tests. Venous blood gas analysis revealed a potential of hydrogen (pH) of 7.36, partial pressure of carbon dioxide (pCO2) of 38 mmHg, partial pressure of oxygen (pO2) of 74 mmHg, and bicarbonate (HCO3) of 25 mmol/L. Abdominal ultrasonography confirmed hepatosplenomegaly with normal echotexture.

On the third day of admission, her condition deteriorated with worsening tachypnea and slight disorientation. Hemoglobin dropped to 4.4 g/dl, leukocyte count increased to 9900/cumm, and platelet count decreased to 8000/cumm. She was transfused with 1 unit of packed red cells and 2 units of platelets. Despite this, her tachypnea worsened, and she exhibited signs of acidotic breathing. Subsequent blood tests showed a hemoglobin level of 7.0 g/dl, leukocyte count of 8500/cumm, and platelet count of 18,000/cumm. Blood gas analysis revealed a pH of 7.1, pCO2 of 27 mmHg, pO2 of 89 mmHg, and HCO3 of 8.8 mmol/L, with a serum lactate level of 12 mmol/L, indicating lactic acidosis.

She was managed with a bicarbonate correction drip, administered as per protocol (0.5 × weight × base deficit). The patient received half the correction over one hour and the remainder over the next 23 hours. Monitoring indicated stable pulses, blood pressure, and urine output and no signs of sepsis. A repeat blood gas analysis showed some improvement (pH: 7.15, pCO2: 22 mmHg, pO2: 101 mmHg, HCO3: 9.7 mmol/L) and a serum lactate level of 10 mmol/L, but there was a worsening of her clinical condition. This was identified as type B lactic acidosis, as type A was ruled out due to the absence of tissue hypoperfusion and sepsis. Consequently, she was switched to oral sodium bicarbonate tablets, as addressing the underlying cause would be the most effective way to improve her clinical condition.

**Figure 1:**
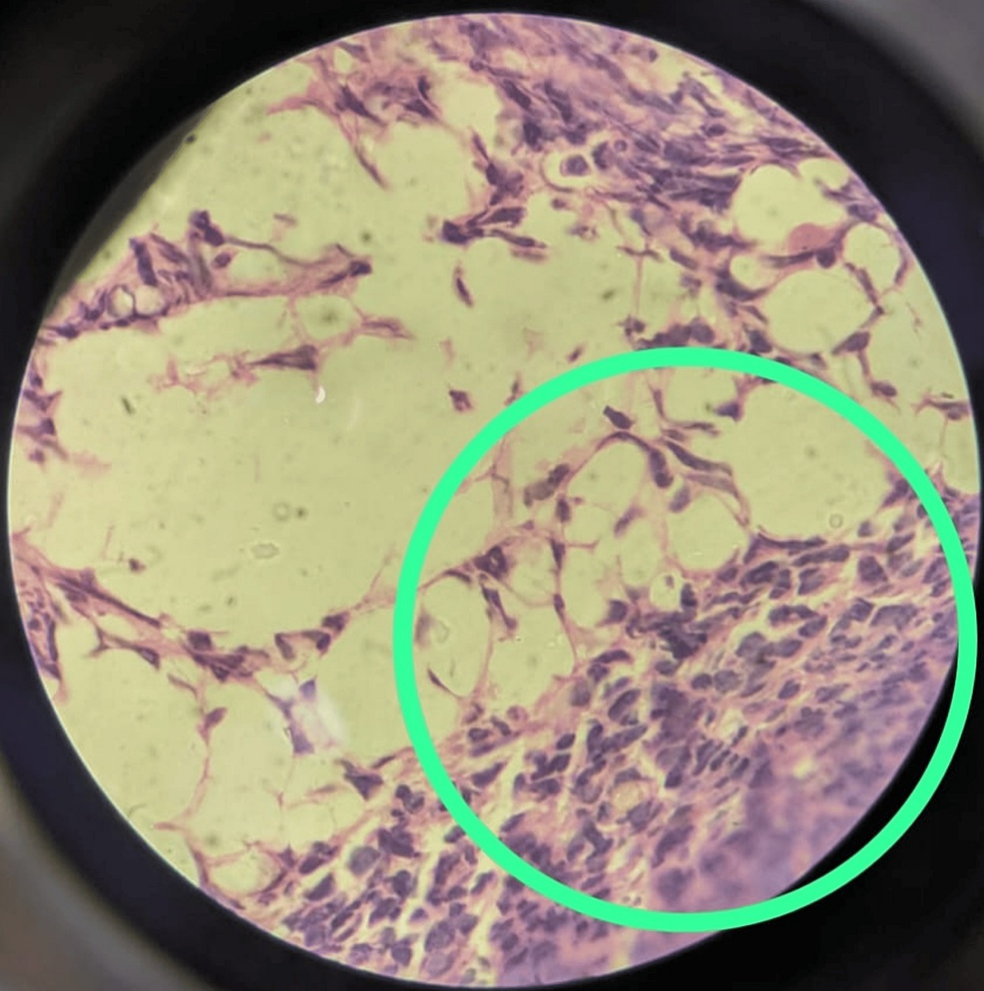
Bone marrow biopsy stained with hematoxylin and eosin under 100× showing the fat marrow and adjacent mononuclear cells of lymphoid origin, effacement of architecture by round blue cells suggestive of acute lymphoblastic leukemia (green circle)

Considering the possibility of hematological malignancy due to bi-cytopenia, bone marrow aspiration and biopsy were performed. The cytological and histopathological evaluations confirmed ALL (Figure 1). Flow cytometry further classified it as precursor B lymphoblastic leukemia (CD10 positive). 

The oncology team initiated induction chemotherapy according to the Berlin-Frankfurt-Münster (BFM)-95 protocol, starting with oral prednisolone (60 mg/m²). Chemotherapy included vincristine (1.5 mg/m² on days 8, 15, 22, and 29), daunorubicin (30 mg/m² on the same days), and L-asparaginase (5000 U/m² on days 12, 15, 18, 21, 24, 27, 30, and 33). Due to the patient's worsening clinical condition, characterized by an altered breathing pattern and sensorium, additional investigations such as a chest radiograph and a computed tomography scan of the brain were performed to rule out organ involvement. Both scans were normal. Attributing the primary cause of deterioration to type B lactic acidosis, the blood gas analysis was closely monitored and showed improvement 30 hours after starting chemotherapy.

**Figure 2:**
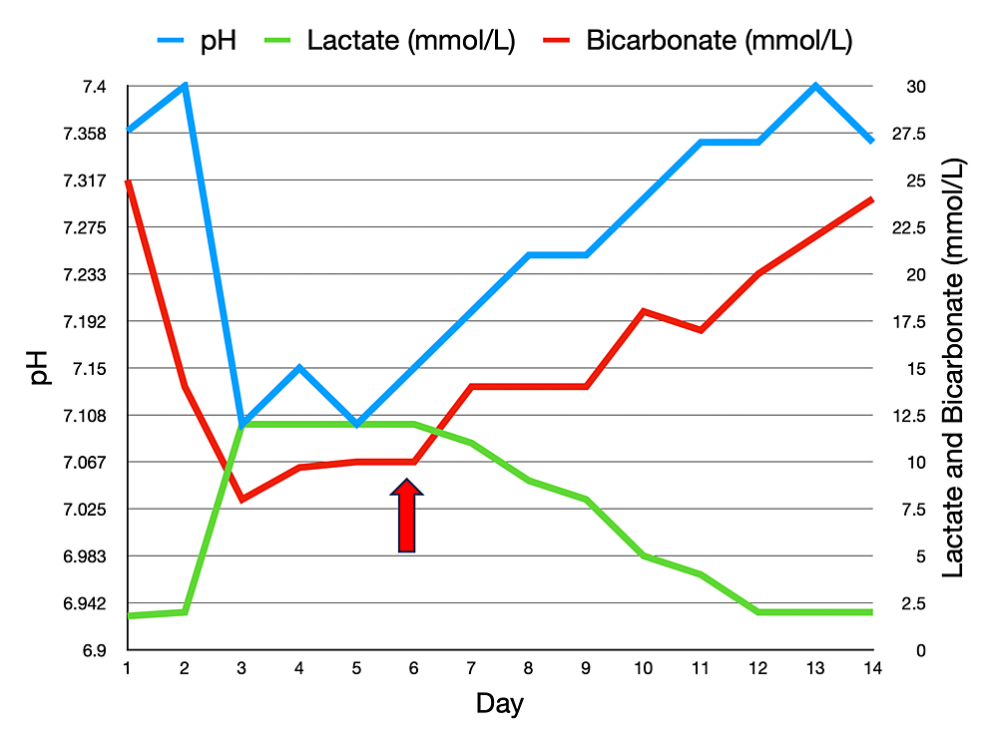
Successive blood gas analyses indicating gradual correction of acidosis, with decreasing serum lactate levels The red arrow indicates the initiation of chemotherapy Image Credit: Chaitanya Kumar Javvaji

Successive blood gas analyses indicated a gradual correction of acidosis, with decreasing serum lactate levels (Table 1, Figure 2). This improvement was reflected in the patient's clinical status, marked by enhanced consciousness and normalized breathing patterns.

**Table 1:** Successive blood gas analyses The values in the round window indicate reference ranges pH: potential of hydrogen; PCO2: partial pressure of carbon dioxide; HCO3: bicarbonate

Day	pH	Lactate (<2 mmol/L)	HCO3 (18-24 mmol/L)	PCO2 (35-45 mmHg)
1	7.36	1.8	25	27
2	7.40	2	14	20
3	7.10	12	8	24
4	7.15	12	9.7	26
5	7.10	12	10	17
6	7.15	12	10	21
7	7.20	11	14	20
8	7.25	9	14	22
9	7.25	8	14	24
10	7.30	5	18	24
11	7.35	4	17	26
12	7.35	2	20	28
13	7.40	2	22	35
14	7.35	2	24	38

The induction phase was continued, and upon completion, the patient's hemoglobin level was 9.2 g/dl, leukocyte count was 6,500/cumm, and platelet count was 9,000/cumm. This prompted the transfusion of 3 units of platelets. A follow-up complete blood count showed improved blood parameters. The sequential laboratory investigations of the patient during the hospital stay are shown in Table 2. The patient was then scheduled for induction phase 2 according to the BFM-95 protocol.

**Table 2:** Sequential laboratory investigations of the patient

Laboratory investigations	Day 1	Day 3	Day 4	Day 7	Day 9	Day 10	Day 12	Day 14	Biological reference range
Hemoglobin	6.3	4.4	7	9.2	9.0	8.8	9.1	9.4	13-15 g/dl
Total leukocyte count	8000	9900	8500	6500	4900	5400	5200	4600	4000-11000/cumm
Platelet	74000	8000	18000	9000	32000	40000	44000	52000	1,50,000-4,50,000/cumm
Urea	16	14	12	14	12		14	14	9-20 mg/dl
Creatinine	0.5	0.5	0.4	0.3	0.4		0.4	0.5	0.6-1.2 mg/dl
Sodium	132	133	136	138	132		138	135	137-145 mmol/l
Potassium	3.6	4	3.9	4.2	4		4.3	4.1	3.5-5.1 mmol/l
Alkaline phosphatase	360	343	337	320	336		349	320	38-126 unit/l
Alanine transaminase	62	46	45	44	48		62	58	<50 U/l
Aspartate transaminase	88	55	73	56	44		56	64	17-59 U/l
Total protein	6.4	6	7	6.4	7.2		7	7.2	6.3-8.2 gm/dl
Albumin	3.1	3.1	3.6	3.4	3.8		3.6	4	3.5-5 gm/dl
Total bilirubin	1.5	1.3	1.4	1.2	1.4		1.2	1.2	0.2-1.3 mg/dl
Unconjugated bilirubin	0.4	0.3	0.3	0.3	0.4		0.3	0.4	0-0.3 mg/dl
Conjugated bilirubin	1.1	1	1.1	0.9	1		0.9	0.8	0-1.1 mg/dl
Globulin	3.3	2.9	3.4	3	3.4		3.4	3.2	2.3-3.5 mg/dl
Activated partial thromboplastin time	30.1	30.6	30.3		30.4			30.2	29.5 control
Prothrombin time	12.6	12.2	12.1		12.2			12.4	11.9 control
International normalized ratio	1.16	1.12	1.14		1.15			1.14	0.8-1.2

## Discussion

Lactic acidosis, though infrequent, stands as a potentially perilous complication in young sufferers of ALL, casting a formidable shadow over the otherwise hopeful landscape of pediatric oncology. Its occurrence poses diagnostic and therapeutic challenges due to its multifactorial etiology and complex underlying mechanisms. In this case report, a 14-year-old female with ALL developed type B lactic acidosis during the course of treatment, highlighting the need for a comprehensive understanding of this metabolic derangement and its management.

Lactic acidosis can be broadly classified into two types: type A and type B. Type A lactic acidosis typically arises from tissue hypoxia or ischemia, leading to anaerobic glycolysis and subsequent lactate production [7]. In contrast, type B lactic acidosis occurs due to underlying medical conditions unrelated to tissue hypoxia, such as hematologic malignancies like ALL [6]. Other causes of type B lactic acidosis include severe infections, liver disease, renal failure, certain medications (e.g., metformin), and inherited metabolic disorders [8].

The mechanisms underlying type B lactic acidosis in pediatric ALL patients are diverse and not fully understood. One contributing factor is the increased glycolytic activity of leukemic cells, known as the Warburg effect, which leads to elevated lactate production even in the presence of adequate oxygenation [9]. The Warburg effect involves a shift in cellular metabolism from oxidative phosphorylation to glycolysis, resulting in excessive lactate production as a byproduct of glucose metabolism [10]. This metabolic shift is a hallmark of cancer cells and is driven by various oncogenes and mutations that alter cellular energy production pathways [11].

Additionally, hepatosplenomegaly, commonly observed in pediatric ALL, can impair hepatic function and decrease lactate clearance, exacerbating lactic acidosis [12]. In the intricate metabolic processes, the liver assumes a pivotal position, executing a critical function in the conversion of lactate to glucose through the mechanism of gluconeogenesis. When the hepatic function is compromised due to leukemic infiltration or other factors, lactate clearance is reduced, leading to accumulation in the blood [13].

Furthermore, the systemic inflammatory response associated with leukemia can contribute to lactic acidosis through various mechanisms, including cytokine-mediated alterations in cellular metabolism and tissue perfusion. Pro-inflammatory cytokines such as tumor necrosis factor (TNF) and interleukins can disrupt mitochondrial function, further promoting glycolysis and lactate production [14]. These cytokines can also induce microvascular dysfunction, impairing tissue perfusion and exacerbating metabolic derangements [15]. Renal dysfunction secondary to leukemic infiltration or tumor lysis syndrome can also impair lactate excretion, further worsening lactic acidosis [16]. The kidneys contribute to lactate clearance by filtering lactate from the blood and reabsorbing it for further metabolism. Renal impairment, therefore, reduces this clearance capacity, leading to elevated lactate levels [17].

Treatment of type B lactic acidosis in pediatric ALL involves addressing both the underlying leukemia and the metabolic derangement. Chemotherapy targeting leukemic cells is essential for controlling the disease and reducing lactate production. Supportive measures, including fluid resuscitation and correction of electrolyte imbalances, are crucial for managing lactic acidosis [18]. Additionally, specific interventions to enhance lactate clearance, such as renal replacement therapy in cases of severe renal impairment, may be necessary [14]. The prognosis of a malignancy with lactic acidosis is multifaceted, influenced by the underlying cause, the promptness of intervention, and the levels of lactate present. Mortality rates align closely with the duration and severity of lactic acidosis. Notably, type B lactic acidosis, as evidenced by studies, underscores a particularly dire outlook [3,19]. Despite aggressive therapeutic approaches, this subtype is associated with an alarming mortality rate surpassing 90% [3].

In this particular case, the patient exhibited symptoms indicative of systemic involvement, notably including abdominal pain, fever, and hepatosplenomegaly. Subsequent laboratory investigations confirmed the presence of severe anemia and thrombocytopenia, consistent with a diagnosis of hematologic malignancy, which was further substantiated by findings from both the bone marrow biopsy report and the flow cytometry report. Despite the administration of aggressive therapeutic measures, encompassing transfusions, antibiotics, soda bicarbonate correction, and comprehensive supportive care, the patient's clinical condition, along with the lactic acidosis, continued to deteriorate. It was only following the initiation of chemotherapy for ALL that gradual improvement, both clinically and biochemically, in lactic acidosis was observed.

Treating the underlying cause of type B lactic acidosis is crucial to prevent recurrence, optimize patient outcomes, and reduce mortality risk. By targeting the primary disease process, clinicians can reverse metabolic disturbances, mitigate complications, and improve organ function, ultimately enhancing the patient's quality of life. This approach necessitates a comprehensive, multidisciplinary care model to ensure prompt diagnosis and effective management, avoiding the masking of symptoms and facilitating better long-term outcomes for patients.

## Conclusions

This report underscores the critical importance of heightened clinical awareness regarding the potential occurrence of type B lactic acidosis in pediatric ALL patients. Early recognition, prompt intervention, and tailored management strategies are essential for improving outcomes and mitigating the morbidity associated with this rare yet potentially life-threatening complication.
